# Supplementary material for: Outcome and factors associated with undernutrition among children with congenital heart disease
Source: PLoS One. 2023 Feb 23;18(2):e0281753. doi: 10.1371/journal.pone.0281753 (PMC9949631; doi:10.1371/journal.pone.0281753)
Supplement: Attachment — Submitted filename: Responses to Reviewers.docx [file pone.0281753.s002.docx]

Reviewer's Responses to Questions

**Comments to the Author**

1. Is the manuscript technically sound, and do the data support the conclusions?

The manuscript must describe a technically sound piece of scientific research with data that supports the conclusions. Experiments must have been conducted rigorously, with appropriate controls, replication, and sample sizes. The conclusions must be drawn appropriately based on the data presented.

Reviewer #1: Yes

Reviewer #2: Partly

2. Has the statistical analysis been performed appropriately and rigorously?

Reviewer #1: Yes

Reviewer #2: Yes

3. Have the authors made all data underlying the findings in their manuscript fully available?

The [PLOS Data policy](http://www.plosone.org/static/policies.action#sharing) requires authors to make all data underlying the findings described in their manuscript fully available without restriction, with rare exception (please refer to the Data Availability Statement in the manuscript PDF file). The data should be provided as part of the manuscript or its supporting information, or deposited to a public repository. For example, in addition to summary statistics, the data points behind means, medians and variance measures should be available. If there are restrictions on publicly sharing data—e.g. participant privacy or use of data from a third party—those must be specified.

Reviewer #1: Yes

Reviewer #2: Yes

4. Is the manuscript presented in an intelligible fashion and written in standard English?

PLOS ONE does not copyedit accepted manuscripts, so the language in submitted articles must be clear, correct, and unambiguous. Any typographical or grammatical errors should be corrected at revision, so please note any specific errors here.

Reviewer #1: Yes

Reviewer #2: No

5. Review Comments to the Author

Please use the space provided to explain your answers to the questions above. You may also include additional comments for the author, including concerns about dual publication, research ethics, or publication ethics. (Please upload your review as an attachment if it exceeds 20,000 characters)

Reviewer #1: The authors performed a cohort study to assess the nutritional status in children with CHD and to assess risk factors for this undernutrition. The topic is interesting and important. The methodology is sound and the manuscript is organized and well written but i have few comments:
1- the manuscript needs good English editing

Response to Reviewer's comment:

We thank you very much for the positive comments and interest in the paper.

We have consulted the manuscript to a native English speaker:

Erik Christopher Hookom, BA, M.Ed, TEFL.

Office of Research and Publication (ORP)

Faculty of Medicine, Public Health and Nursing, Universitas Gadjah Mada

Administration Building 2nd Floor

Phone: +62 274 560300 ext 205

Email: echookom@gmail.com

We have carefully revised the manuscript to address the errors and grammatical mistakes throughout the paper.

2- what were the primary and secondary outcomes? Please add them at the end of the method section

Responses to Reviewers:

Thank you very much for your suggestion. We have included the primary and secondary outcome at the end of the method section. We added *“The primary outcome of this study was the outcome of undernutrition among children with CHD. The secondary outcome were factors associated with undernutrition among children with CHD.”*

3- what is the power of the study? And how did you estimate the sample size?

Responses to Reviewers:

We have tried to calculate the estimated sample size using this formula below with 80% of power and the number of subjects included in our study exceeded the estimated sample size.

The prevalence of malnutrition in children with congenital heart disease based on a previous study by Batte et al. 2017 is 31.5% - 45.4%.

$$Sample size= \frac{z^{2} x p \left( 1-p \right)}{c^{2}}$$

$$z=z value z=z value \left( 1.96 for 95\% confidence level \right)$$

$$p=proportion of malnutrition in children with CHD (estimated 31\%)$$

$$c=margin of errors \left( expressed in decimal \right)(0.05)$$

$$Sample size= \frac{{1.96}^{2} x 0.31 \left( 1-0.31 \right)}{{0.05}^{2}}$$

$$Sample size=329$$

Reviewer #2: This study prospective investigated the burden of congenital heart disease (CHD) in Indonesia and nutritional status among children with CHD in one single center. The authors identified some risk factors associated with undernutrition. The case number in this study is large. I have some comments as below.

Major comments:
1. The definitions of underweight, stunting and wasting in methods were repetitive. Consider shorten the paragraph. The same problem occurred in discussion. The discussions of underweight, stunting and wasting in three separated paragraphs are redundant and repetitive.

Responses to Reviewers:

We thank you very much for the positive comments, suggestion, and interest in the paper.
We have shortened the paragraph into *“The nutritional status was determined using criteria which were derived from the standards of the World Health Organization (WHO). Undernutrition included underweight, stunting and wasting. “*

2. The discussion is lengthy. Consider shorten the paragraph to half and avoid repetitive descriptions on each finding.

Thank you very much for your suggestion. We have revised and shorten the discussion section.

3. Some factors in table 2 should be defined, such as “syndrome” and “delayed diagnosis”. What diagnosis was included in syndrome or how to make a diagnosis of syndrome should be described.

Thank you very much for your suggestion. We have included this sentence into the Methods:

*“Presence of genetic syndrome was defined when patients were diagnosed as having any congenital dysmorphic syndrome. The diagnosis of syndrome was defined based on clinical appearance of the patients”.* The most common syndrome was Down syndrome.

“*Delayed diagnosis was defined based on their pathologic type: cyanotic CHD and acyanotic CHD. Delayed diagnosis in acyanotic CHD was defined at an age where elective cardiac repair should have already been performed or in case immediate treatment was indicated because of the patient hemodynamic status. Patient with delayed diagnosis in cyanotic CHD were defined as newborns discharged from their birth clinic or hospital without a CHD diagnosis”.*

4. Figure 1 is missing.

Thank you. We have submitted the figure 1 in different file.

5. Why the risk factors of underweight, stunting and wasting are different? In-depth discussion is encouraged.

Thank you very much for raising this. The presence of undernutrition was associated with increased risk of death among children with CHD. Some of our findings were in line with previous references while some were not. For example, it is interesting to note in our study that children with cyanotic CHD present as a risk factor in both episodes of wasting and stunting. This is in line with studies from Chinawa et al. in Nigeria, Basheir et al. in Egypt, Zhang et al. in China, and Okoromah et al. in Nigeria that stunting or wasting episodes are found in children with cyanotic CHD. However, some studies noted that only stunting was associated with cyanotic CHD, while others stated that only wasting was associated with cyanotic CHD (6,7,15,16). Both stunting and wasting are indicators of chronic malnutrition. Chronic hypoxia from right to left lesion with possible prolonged pulmonary hypertension seen in cyanotic CHD provides direct and indirect effects on reduced serum hormone insulin-like growth factor I (IGF-I) that can cause impairment of bone center and eventually impair nutritional status and linear growth (17). This could explain why in our study, cyanotic CHD was not a risk factor for underweight which marks acute undernutrition. Therefore, in this paper, we choose to mention each condition as a separate risk factor.

Minor comments:
1. “Multivariate” logistic regression is more suitable than “Multivariable” logistic regression.

Thank you very much for your suggestion. We have revised it in abstract and methods section.

2. Suggest replace “Syndrome” in abstract as a more specific term.
Thank you very much for your suggestion. We have revised as suggested.

3. Abbreviations should be listed below each table.
Thank you very much for your suggestion. We have listed the abbreviation below each table in the manuscript.

4. Extensive English editing is needed.

Thank you very much for your suggestion. We have consulted the manuscript to a native English speaker:

Erik Christopher Hookom, BA, M.Ed, TEFL.

Office of Research and Publication (ORP)

Faculty of Medicine, Public Health and Nursing, Universitas Gadjah Mada

Administration Building 2nd Floor

Phone: +62 274 560300 ext 205

Email: echookom@gmail.com

We have carefully revised the manuscript to address the errors and grammatical mistakes throughout the paper.

6. PLOS authors have the option to publish the peer review history of their article ([what does this mean?](https://journals.plos.org/plosone/s/editorial-and-peer-review-process#loc-peer-review-history)). If published, this will include your full peer review and any attached files.


If you choose “no”, your identity will remain anonymous but your review may still be made public.


**Do you want your identity to be public for this peer review?** For information about this choice, including consent withdrawal, please see our [Privacy Policy](https://www.plos.org/privacy-policy).

Reviewer #1: No

Reviewer #2: No

[NOTE: If reviewer comments were submitted as an attachment file, they will be attached to this email and accessible via the submission site. Please log into your account, locate the manuscript record, and check for the action link "View Attachments". If this link does not appear, there are no attachment files.]

While revising your submission, please upload your figure files to the Preflight Analysis and Conversion Engine (PACE) digital diagnostic tool, <https://pacev2.apexcovantage.com/>. PACE helps ensure that figures meet PLOS requirements. To use PACE, you must first register as a user. Registration is free. Then, login and navigate to the UPLOAD tab, where you will find detailed instructions on how to use the tool. If you encounter any issues or have any questions when using PACE, please email PLOS at [figures@plos.org](mailto:figures@plos.org). Please note that Supporting Information files do not need this step.
